# On the Causes and Morbid Anatomy of Mental Diseases

**Published:** 1855-01-01

**Authors:** John Webster

**Affiliations:** Fellow of the Royal College of Physicians.


### ON THE CAUSES AND MORBID ANATOMY OF MENTAL DISEASES.

BY JOHN WEBSTER, M.D., F.R.S.

*Fellow of the Royal College of Physicians.*

(Continued from p. 637, No. XXVIII.)

No. 41.—M., æt. 65. In hospital five months.—*Head:* Skull-cap remarkably light and thin. On removal, much fluid blood flowed from divided vessels. Slight opacity of arachnoid; pia mater infiltrated with turbid fluid; cerebral vessels generally full, and substance of brain firm.—*Chest:* Firm adhesions of both lungs, and also numerous tubercles.

No. 42.—M., æt. 57. In hospital twenty-nine years and four months.—*Head:* Vessels, both external and internal, unusually empty. Convolutions partially shrunken, showing large intervals filled with perfectly limpid fluid, infiltrated in cellular tissue of pia mater. Between one and two ounces of serum in each lateral ventricle; much fluid about velum, and large quantity in base of skull, after removal of brain.—*Chest:* Some inconsiderable old adhesions of lungs, with spots of inflammation in each organ; also hepatisation. Heart greatly enlarged, of compact, dense structure, and cavities filled with coagula.

No. 43.—M., æt. 38. In hospital ten years, eight months, and two weeks.—*Head:* General infiltration of pia mater, covering hemispheres. Upwards of an ounce of fluid in each lateral ventricle, and much also about velum. *Chest:* Right lung partially adherent; contained tubercles, and numerous excavations. Left lung also adherent, and tuberculated.—*Abdomen:* Peritoneum, in whole extent, showed tubercular degeneration; viscera universally adherent to each other.

No. 44.—F., æt. 49. In hospital five days.—Body fat, subcutaneous adipose tissue being, in some situations, nearly two inches thick.—*Head:* Arachnoid thin and transparent. Moderate effusion of pia mater. Cerebral substance firm. Ventricles contained more than an ounce of clear serum; also small quantity in base of skull after removal of brain.—*Abdomen:* Uterus contained a large fibrous tumour attached to substance, whereby os tincæ was tilted upwards.

No. 45.—F., æt. 51. In hospital two months and three weeks.—*Head:* Dura mater adhered firmly to skull-cup. Arachnoid transparent. Pia mater so full of blood as to impart general dark-blue hue to convex surface of hemispheres. Convolutions shrunken. Cerebral substance soft and easily torn.—*Chest:* Old adhesions of both pleuræ. Numerous tubercles and punctured cicatrices in both lungs. Posterior part of left lung dark coloured, heavy, and friable.—*Abdomen:* Uterus contained ten fibrous tumours, the size of marbles.

No. 46.—M., æt. 31. In hospital eight months and a-half.—*Head:* Vessels generally empty. Arachnoid slightly opaque. Serous infiltration of pia mater covering hemispheres. About an ounce of fluid in each lateral ventricle. Cerebral substance generally softish, especially in forniix and adjacent parts.—*Chest:* Both lungs tuberculated with purulent excavations.—*Abdomen:* Acute general peritonitis, with agglutination of viscera, and copious puriform effusion. Ulcerations of intestines in various portions; whilst mucous, muscular, and serous coats had given way in some places, but a process of repair seemed in others to have made progress.—Body emaciated.

No. 47.—F., æt. 61. In hospital three months and two weeks.—*Head:* External vessels quite empty, internal more full. Infiltration of pia mater. Increased quantity of fluid in lateral ventricles.—*Chest:* Left lung tuberculated with purulent excavations. Also recent pleurisy. Tubercles likewise in right lung.

No. 48.—F., æt. 46. In hospital twelve months.—*Head:* Slight infiltration of pia mater. About an ounce of fluid in each lateral ventricle. Partial opaque thickening of arterial trunks at base of brain.—*Chest:* Old adhesions of lungs; did not collapse, broke down on pressure, and loaded with fluids. Bronchial tubes reddened, and filled with frothy mucus.—*Abdomen:* Liver adhered to diaphragm. Kidneys only one-third natural size, lobulated externally, and capsule adhered firmly to substance. Structure normal.

No. 49.—M., æt. 64. In hospital three months.—*Head:* Very firm adhesions of dura mater to cranium, which was torn on skull-cap being detached. Vessels of bone and membrane filled with blood. General infiltration of pia mater. Between one and two ounces of serum in both lateral ventricles; also much fluid in base of skull, after removal of brain. Vessels of pia mater and cerebral substance extremely turgid, with numerous and large bloody points on cut surfaces.—*Chest:* Between one and two pints of reddish fluid in left pleura. Circumscribed empyema, containing pus at lower part of cavity. Lung inflamed, of livid black colour, apparently in progress of mortification, and exhaling characteristic fœtor. Neighbouring pulmonary substance bright scarlet.

No. 50.—M., æt. 27. In hospital four months.—*Head:* Skull-cap light. Dura mater nearly white. Pia mater full of blood. Cerebral substance moderately firm and vascular. Considerable serous infiltration between convolutions, and at base of skull after removal of brain.—*Chest:* Both lungs tuberculous; left adherent to ribs, and excavated by large cavern. Right lung consolidated nearly throughout, and exhibited numerous vomicæ.—*Abdomen:* Numerous ulcers in ileum. Mesenteric glands large, and contained tuberculous matter. —P.S. Patient extremely emaciated.

No. 51.—F., æt. 27. In hospital one month.—*Head:* Skull-cap light and thin, with frontal portion particularly flat. Vessels of dura mater full of blood. Opacity of arachnoid. Pia mater converted by serous infiltration into loose spongy-like tissue. Cerebral substance soft. Ventricles contained about three ounces of serum. Foramen of Monro unusually large. Much fluid at base of skull after removal of brain.—*Chest:* Heart small. Old adhesions of left lung; superior lobe puckered and tuberculous. — *Abdomen:* Cavity distended by reddish brown serum, containing numerous flasks of lymph. Great omentum thickened. Convolutions of intestines matted together. Peritoneum pink-coloured. Perforation of ileum near cæcum through which contents had escaped into peritoneal cavity. Mucous surface of intestine covered by continuous layer of lymph for three inches; and numerous elevations of Pyer's glands, as if enlarged. In this portion of canal seven or eight pieces of comb commonly worn in the hair by females were found. They appeared quite black, and like needles, whilst two bits had perforated walls of intestine near greatest aperture. Ovaries large, dark coloured, and contained clots of blood.

No. 52.—F., æt. 61. In hospital three weeks. *Head:* Dura mater adhered firmly to bone, whereby membrane was torn on separation. Arachnoid transparent, but pia mater gorged with blood. A few drams of clear serum in ventricles. Cerebral substance firm.—*Chest:* Left lung most extensively tuberculous, and whole superior lobe excavated into vomicæ. Effusion of soft yellow lymph in pleural sac, with adhesions. Right lung similarly diseased.—*Abdomen:* Two circular ulcers on ileum, and mesenteric glands enlarged.

No. 53.—F., æt. 21. In hospital one month.—*Head:* Skull-cap dense and heavy. Arachnoid transparent, but raised from surface of brain by large quantity of clear serous fluid infiltrated into areolar tissue of pia mater. Cerebral substance soft. Ventricles distended by serum, and much serous fluid at base of skull after brain was removed.

No. 54.— F., æt. 60. In hospital three months and one week. *Head:* Effusion into pia mater. Posterior cerebral convolutions shrunken. Arachnoid transparent. Brain firm and white.—*Chest:* Heart large, soft, and flabby. Valves all thickened and opaque; two of aortic semilunars being united into one piece. A congenital malformation. Left pleura universally adherent. Cavity containing nearly two pints of reddish brown serous fluid, with lymph in lower third. Lung compressed. Cretaceous bodies in right lung; largest, size of a pea. Lining membrane of trachea red.—*Abdomen:* Kidneys one third natural size. Cortical substance strongly adherent to fibrous capsule. Surface of organ rough and granular.

No. 55.—M., æt. 45. In hospital fourteen years and ten months.—*Head:* Dura mater deeply tinged yellow colour. Arachnoid transparent, with small quantity of serous effusion, also of yellow hue. About two ounces of serum in lateral ventricles. Both corpora striata much less prominent than natural, and appeared to have undergone absorption.—*Chest:* Numerous old adhesions in both pleuræ. Posterior part of right lung deep livid colour, friable, and emitted offensive odour of putrefaction. Valves of heart thickened and opaque.—*Abdomen:* Large effusion of yellow serum. Liver small and knobbed; with masses of medullary cancer, varying in size from filbert to large walnut, scattered throughout. Gall bladder empty. Spleen adhered firmly to abdominal parietes.

No. 56.—F., æt. 26. In hospital five days.—*Head:* Dura mater adhered firmly to bone, leaving shreds attached. Arachnoid dry. Pia mater infiltrated by serum; and raised from adjacent cerebral convolutions, which, in many places, seemed shrunken and atrophied. Cerebral vessels full of blood, with tinged cut surfaces of a reddish hue. About two ounces of clear fluid in ventricles; and also much at base of skull. Right posterior clinoid process of sphenoid bone projected about half an inch beyond its fellow, smooth, crusted with cartilage, and in any other situation might have been taken for an exostosis.

No. 57.—F., æt. 37. In hospital fourteen months and one week.—*Head:* Skull-cap thick and heavy. Vessels of brain and membranes turgid throughout. Infiltration of pia mater, and slightly increased effusion of fluid in lateral ventricles.—*Chest:* Superior lobe of left lung extensively congested on posterior aspect; with partial hepatization. Similar change in upper lobe of right lung, and an excavation, containing dark, offensive fluid.

No. 58.—M., æt. 43. In hospital six months and three weeks.—*Head:* Arachnoid sac dry. Membrane white and opaque. Cerebral substance soft and white. Convolutions flattened. Lateral ventricles distended by clear serous fluid.—*Chest:* Large aneurismal sac of aortic arch, adherent to sternum, rent into pericardium, which was distended by about one and a-half pound of deep-black coagulated blood. Semilunar and mitral valves thickened and hard. Heart soft. Lungs bulged from cavity on removal of sternum, and posterior parts broke readily under pressure.—*Abdomen:* Liver hard, and pressed down.—

wards to umbilicus. Fibrous capsule of kidneys adhered with preternatural firmness, portions of cortical substance being torn in attempting separation.

No. 59.—M., æt. 63. In hospital three months and three weeks.—*Head:* Slight serous infiltration of pia mater. About half a teaspoonful of blood in right middle fossa of basis cranii. Coagulum adhered to dura mater; but no breach observed on cerebral surface, nor any appearance of external injury. An ounce, at least, of limpid fluid in each lateral ventricle, and much remained in base of skull after removal of brain.—*Chest:* Universal firm adhesions of left lung to cavity. Right partially hepatized, with lower portion congested and softened. Heart beyond normal size, so that pericardium in contact with parietes about an inch. Left ventricle very thick and firm.

No. 60.—F., æt. 42. In hospital six months and three weeks.—*Head:* Skull-cap extremely thick, heavy, and dense in structure; cancellous texture entirely disappeared. Vessels of dura mater full of blood. No arachnoid sac over left hemisphere; several flakes of extravasated blood, some of considerable size. Brain small but firm, and vessels moderately full. Slight extravasations of blood upon surface of cerebellum, and at base of skull.—*Chest:* Heart small and contracted. Lungs deep-blackish hue, and lining membrane of trachea congested.—*Abdomen:* Large ovarian cyst in pelvic cavity. Right ovary almost disappeared. Left occupied by five smaller cysts; and all contained lympid fluid.

No. 61.—M., æt. 29. In hospital six months.—*Head:* Opaque white spots in arachnoid. Vessels of pia mater and cerebral substance full of blood. Ventricles contained considerable amount of clear serous fluid. Septum lucidum torn, and in shreds.—*Chest:* Universal firm adhesions on right side of thorax. Lung everywhere tuberculous, also hepatized. Left pleural sac contained about two pints of sero-purulent fluid. Left lung coated with thick lymph, and both organs contained numerous cavities.

No. 62.—F., æt. 63. In hospital two days.—*Head:* Skull-cap dense, heavy, and shallow. Pia mater converted into loose, spongy texture by serous effusion; especially along superior longitudinal fissure. Arachnoid white and firm. Ventricles contained a few drachms of lympid fluid.—*Chest:* Old adhesions of pleuræ, with two scattered cretaceous deposits in pulmonary texture. Both heart and lungs pushed upwards by abdominal viscera so as to diminish considerably thoracic cavity.—*Abdomen:* Large ovarian cyst occupied whole of pelvis. Solid white fibrous tumour, about size of walnut, upon wall and in situation of ovary. Ureters dilated. Kidneys large, dark-coloured, and full of blood.

No. 63.—M., æt. 35. In hospital six weeks.—*Head:* Not a drop of blood flowed on dividing integuments and exposing cranium. Vessels of dura mater not unusually full; but those of pia mater and cerebral substance turgid to minutest ramifications. Arachnoid, over entire cerebral surface, slightly and partially opaque, being of milky colour in intervals of convolutions. Slight infiltration of pia mater. Each lateral ventricle contained about an ounce of pellucid fluid.—*Chest:* Pleuræ in both cavities inflamed throughout, and each contained nearly two pints of sero-purulent fluid, with some flakes of soft fibrin. Lungs consolidated, and exhibited congestive stage of pneumonia. Left lung connected to thorax by old adhesions.

No. 64.—F., æt. 65. In hospital five months.—*Head:* Bones of skull unusually thick and heavy. Arachnoid partially opaque. Pia mater infiltrated. Lateral ventricles distended by large quantity of limpid fluid. Septum lucidum torn and attenuated. Cerebral substance somewhat congested, and bloody points on cut surfaces large and numerous. Much fluid in base of skull after removal of brain.

No. 65.—F., æt. 35. In hospital seven months and three weeks.—*Head:* Not a drop of blood in exterior coverings of cranium. Vessels of brain and membranes turgid everywhere. Thickening and slight milky opacity of arachnoid over hemispheres. Pia mater considerably infiltrated. Each lateral ventricle contained at least an ounce of perfectly limpid fluid, and much remained in base of skull after removal of brain.—*Chest:* Pulmonary substance of left lung consolidated, dull brownish colour, and several small suppurations.

No. 66.—F., æt. 18. In hospital seven weeks. *Head:* Skull-cap adhered firmly to dura mater. Arachnoid opaque. Considerable effusion of clear fluid into pia mater, occupying intervals between shrunken convolutions. Cerebral substance full of blood, and serum in lateral ventricles.—*Chest:* Trachea filled with light-brown, extremely offensive fluid. Gangrenous odour prevailed on removing sternum. Right lung extensively mortified, and similar changes, but to less extent, in left pulmonary tissue.—*Abdomen:* Stomach enormously distended with light, chocolate-coloured fluid. Mucous membrane of viscus soft, and almost decomposed. Slight intus-susception of ilium.

No. 67.—M., æt. 38. In hospital one year.—*Head:* Internal vessels full. Partial milky opacity of arachnoid on cerebral hemispheres, particularly between the two lobes, where membrane was loose; and left large sub-arachnoid space. Slight infiltration of pia mater. Increased deposition of fluid in ventricles, and very large quantity in base of skull, after removal of brain.—*Chest:* A few old adhesions of lungs to cavity of thorax.

No. 68.—F., æt. 33. In hospital seven months and three weeks.—*Head:* Skull-cap thick and heavy. General infiltration of pia mater. About one and a-half ounce of perfectly limpid fluid in each lateral ventricle; and much fluid also remaining at basis cranii after removal of brain.—*Chest:* Slight old adhesions at upper lobe of left lung to cavity.

No. 69.—M., æt. 32. In hospital six months.—*Head:* Skull-cap shallow, and very firmly adherent to dura mater. Slight opacity of arachnoid. Pia mater infiltrated by serum. Convolutions shrunken, and cerebral substance atrophied. Lateral ventricles greatly distended, and contained about four ounces of clear serous fluid.—*Chest:* Right lung congested, friable, solid, and contained no air.

No. 70.—M., æt. 44. In hospital two weeks.—*Head:* Integuments infiltrated by sero-purulent fluid; and skin of upper eyelids partially sloughed. Pericranium stained of red colour, and vessels congested with blood. Dura mater of reddish hue, on both surfaces, from congested bloodvessels. Pia mater infiltrated with turbid serum. Ventricles distended. Under middle lobe, at base of skull, about half an ounce of blood, forming a clot, had escaped from a superficial cerebral vein. Adhesion of cerebral hemispheres along superior longitudinal sinus, so strong as only separable by knife. Encephalic contents full of blood.—*Chest:* Old and firm adhesions of right pleura.—*Abdomen:* Strong adhesion, of old date, between intestines.

No. 71.—M., æt. 38. In hospital one year and ten weeks.—*Head:* General reddish discolouration of pia mater covering hemispheres, from transudation of blood; membrane also considerably infiltrated.—*Chest:* Left lung connected to thorax by recent general adhesions; its surface being covered with thin adventitious layer of fibrin. Posterior portion partly congested and hepatized, with a small collection of pus. Right lung also congested, and partially hepatized.

No. 72.—M., æt. 18. In hospital twelve days. Soft structures on back of hand, fore and upper arm generally disorganized by violent imflammation; with extensive suppuration and sloughing of skin and cellular tissue in two latter situations.—*Head:* Bloody points on cut surfaces of medullary substance numerous. Increased quantity of fluid in ventricles.—*Chest:* Right lung distended, with congestion. Left collapsed.

No. 73.—F., æt. 39. In hospital thirty-two days.—*Head:* Dura mater adhered strongly to skull-cap. Arachnoid partially opaque. Pia mater infiltrated by serous effusion. Cerebral convolutions atrophied, and leaving considerable spaces between. Ventricles much distended, and contained about three ounces of limpid fluid: cerebrum small.—*Chest:* Right lung universally adherent to parietes.

No. 74.—M., æt. 24. In hospital six days.—*Head:* Vessels of brain and membranes turgid. General infiltration of pia mater. About one and a-half ounce of fluid in lateral ventricles, and much remained at base of skull after removal of brain.—*Chest:* Recent acute inflammation of pleuræ covering each lung, with copious effusion of soft yellow fibrin. Pulmonary substance in highest state of vascular congestion, and adventitious deposit in texture, but without any trace of suppuration.

No. 75.—M. æt. 39. In hospital six weeks.—*Head:* Cerebrum small in size. Slight and partial opacity of arachnoid. Considerable effusion of serous fluid into pia mater, which occupied spaces between shrunken convolutions, like a sponge. Ventricles contained a considerable amount of serous fluid. Cerebral substance red, from injection of bloodvessels. Patient died of erysipelas.

No. 76.—F., æt. 48. In hospital twenty-two years and two months.—*Head:* Arachnoid covering hemispheres thickened and opaque. Pia mater greatly infiltrated; and about an ounce of limpid fluid in each lateral ventricle. Brain soft.—*Chest:* Heart large and flabby: dropsical effusion both in thorax and abdomen. Patient having died of anasarca.

No. 77.—E., æt. 46. In hospital three weeks.—*Head:* Slight effusion under arachnoid. Convolutions flattened, and ventricles filled with limpid serum.—*Chest:* Some old adhesions of right pleura. Left lung converted into soft, black, and nearly airless mass, with portion of middle broken down, and containing a reddish-brown fluid.—*Abdomen:* Large fibrous tumour in uterus.

No. 78.—M., æt. 44. In hospital twelve years.—*Head:* Skull-cap adhered so firmly to dura mater that it was separated with difficulty. Parietal layer of arachnoid red, from injection of vessels in dura mater with blood. Soft, and continuous layer of fibrin extended from base of skull to fossa of cerebellum. Ventricles distended by clear, limpid serum. Arachnoid partially opaque, with considerable amount of fluid under membrane.—*Chest:* From three to four pints of sero-purulent fluid in right pleural sac, whereby lung was compressed. Left lung contained tuberculous matter, also cavities, and adhered to thoracic parietes.—*Abdomen*: Ilium congested, and small ulcers near junction to cæcum.

No. 79.—M., æt. 33. In hospital six weeks.—*Head:* Dura mater adhered so firmly to bone as to be torn into shreds during separation. Arachnoid universally of milky-white colour, and opaque; membrane raised from convolutions by much serous infiltration, converting pia mater into a spongy texture. Cerebral substance red through injection of bloodvessels. Ventricles distended by clear fluid. Choroid plexus empty of blood, white, and colourless.—*Chest:* Both lungs contained cretaceous tubercles; and at apex of left, puckered cicatrix, upon which several small, hard, earthy masses projected.

No. 80.—M., æt. 39. In hospital six months and one week.—*Head:* Much dark and rather thick blood escaped on dividing scalp. Arachnoid over whole extent of hemispheres opaque and thickened. Pia mater considerably infiltrated, and adhered closely, as also extensively, to brain; this covering of hemispheres looked like spongy substance. Wherever sections were made, copious effusion of infiltrated fluid supervened. About two ounces of perfectly limpid fluid in each lateral ventricle. Internal vessels of brain turgid.—*Chest:* Some old adhesions of pleuræ. Heart hypertrophied; with sides of right ventricle thick and dense.

No. 81.—F., æt. 25. In hospital two months.—*Head:* Cranium thick, heavy, and vascular. Vessels of brain and membranes turgid. Bloody points, on cut cerebral surfaces, large and numerous. Moderate infiltration of pia mater. About an ounce of limpid fluid in each lateral ventricle, and much also remained in base of skull after removal of brain.—*Chest:* Recent adhesions of right lung; organ impervious to air, solid, inflamed, and contained tubercles. Left lung at its lower part also in congestive stage of pneumonia.

No. 82.—F., æt. 66. In hospital eight months and a-half.—*Head:* Dura mater adhered very firmly to skull-cap, being torn into shreds upon separation of bone. Brain small, soft, and anterior lobe remarkably flat.—*Chest:* Gangrene of left lung, posterior part being of deep livid black colour, soft and friable, and emitting a most offensive odour. Valves of heart opaque and thickened.—*Abdomen:* Congestion of mucous membrane of ilium. Kidneys large.

No. 83.—F., æt. 65. In hospital two weeks.—*Head:* Skull-cap adhered so firmly to dura mater that membrane was torn to shreds upon removal of bone. Arachnoid partially opaque. Pia mater converted into loose spongy mass, occupying wide interspaces between convolutions, which were atrophied and shrunken. Moderate effusion of clear fluid in ventricles, and much at base of skull after removal of brain.—*Chest:* Sac of pericardium distended by several ounces of reddish-brown serum, and inner surfaces covered by thick layer of soft fibrin. Muscular substance soft, and tore readily. Circumscribed cavity in sac of left pleura contained a pint of serous fluid, and lymph, in parts half an inch thick, separated this cavity from lower portion of pleura.—*Abdomen:* Uterus contained one fibrous tumour the size of a walnut.

No. 84.—F., æt. 51. In hospital four months.—*Head:* Skull-cap thick, dense, and heavy, with extremely firm adhesions of dura mater, infiltration of pia mater, and about an ounce of fluid in lateral ventricles.—*Chest:* Firm old adhesion of right lung, with tubercular matter and cretaceous deposit in substance.—*Abdomen:* Close adhesion of diaphragm to convex surface of liver. Numerous elongated adhesions of liver to stomach and arch of colon. Soft but tough fibrous tumour in wall of uterus.

No. 85.—M., æt. 30. In hospital four months and one week.—*Head:* Internal vessels all turgid, those of pia mater being injected with blood to minutest ramifications. Pia mater also considerably infiltrated, and adherent so firmly to surface of brain that portions of grey substance were torn away on being detached. This state also general in cerebrum and cerebellum. Lateral ventricles much distended, opening of communication under fornix large and circular, with at least an ounce of fluid in each cavity, of which a quantity also remained at base of skull after removal of brain.

No. 86.—M., æt. 71. In hospital fifty-one years and ten months.—*Head:* Upon skull-cap being detached bloodvessels, passing into cranial bones at sutures, so full and numerous as to give dura mater in those situations a bright redness, contrasting strongly with paleness of membrane generally noticed. Some infiltration of pia mater. Increased quantity of fluid in ventricles. Cerebral substance rather soft.

No. 87.—M., æt. 9. In hospital thirty-four days.—*Head:* Dura mater adhered so firmly that it was difficult to detach skull-cap. Vessels of bone all filled with blood. Channels for superior longitudinal and lateral sinuses strongly marked. Cranial contents filled cavity so very closely, that tense dura mater bulged over sawn edge of bone. Not the slightest moisture between membrane and its contents, which adhered closely to latter. Convolutions perfectly flattened over whole surface of brain. Large effusion of blood in substance of left cerebral hemisphere at level of corpus callosum, and parallel with lateral ventricle, but did not communicate; quantity nearly four ounces, and recently coagulated. There was also about an ounce of watery fluid tinged with blood. In exterior lobe of brain, near extravasation, a small narrow cavity, about an inch in length, also existed, lined by thin smooth covering, surrounding substance being of dull buff colour.—*Chest:* Lungs did not collapse. Heart and pericardium much larger than usual, and in direct contact with sternum. Left ventricle hypertrophied.—*Abdomen:* Liver depressed about three inches below cartilaginous border of thorax.

No. 88.—M., æt. 52. In hospital two months and three weeks.—*Head:* Internal vessels turgid. Slight partial opacity of arachnoid. Considerable general infiltration of pia mater. Lateral ventricles greatly distended, from containing nearly four ounces of perfectly pellucid fluid. Septum lucidum increased in depth by distension, and so thin as if about to give way. Two cavities communicated directly by circular aperture.— *Chest:* Close old adhesions of both lungs. Muscular substance of heart flaccid, and rather pale.

No. 89.—M., æt. 55. In hospital thirty-three years and ten months.—*Head:* Dura mater adhered so firmly to bone that membrane was torn into shreds on separating skull-cap. Arachnoid transparent. Slight effusion of serous fluid into pia mater between convolutions, which were partially atrophied. Cerebral substance firm and white. Lateral ventricles contained nearly three drachms of slightly turbid serum. Vessels upon surface of brain full of fluid blood. Patches of soft, yellow, recently-effused fibrin, about the size of a shilling, in arachnoid sac, at base of brain.— *Chest:* Dropsical effusion in pleural sacs. Pericardium similarly distended. Heart twice natural size. Valves slightly thickened. Both lungs inflamed, with recently-effused yellow fibrin in some parts, other portions being soft, pliable, and of a deep-black hue.—*Abdomen:* Liver dark-coloured, and veins full of blood. Spleen puckered on surface, as if by a cicatrix; interior friable, and of light reddish-brown or yellow colour. Kidneys about half normal size. Capsules adhered firmly to outer surface; rough and granular. Right kidney full of blood, and dark-coloured.

No. 90.—F., æt. 38. In hospital five months.—*Head:* Skull-cap heavy, compact, and without diploe. Dura mater red from injection of bloodvessels. Arachnoid milky white. Convolutions flattened, compressed, and bulged over sawn edge of cranium. Ventricles much distended by three to four ounces of limpid serum. Vessels of cerebrum full of blood, and much fluid in basis cranii after removal of brain.—*Chest:* Right pleura and lung inflamed. Organ heavy; pulmonary substance dark-coloured, friable, and easily breaking down on pressure.—*Abdomen:* Small fibrous tumour in uterus.

No. 91.—F., æt. 40. In hospital twelve months.—*Head:* Slight opacity of arachnoid over cerebral hemispheres. Infiltration of pia mater in same situation. About eight or ten drachms of perfectly pellucid fluid in lateral ventricles. Fornix elevated, and so made foramen of Monro a direct communication between two cavities.—*Chest:* Both lungs congested, contained but little air, and easily broke down under pressure. Upper lobe of left lung contained tubercles, with purulent excavations.

No. 92.—M., æt. 50. In hospital nineteen years and eight months.—*Head:* Dura mater adhered so firmly, that shreds were torn away by removing skull-cap. Slight infiltration of pia mater on cerebral hemispheres. About an ounce of limpid fluid in each lateral ventricle. Bloody points on cut surfaces of brain numerous.—*Chest:* Heart considerably enlarged, and valves diseased; aortic having an osseous deposit. Right pleura inflamed, and contained about a pint of slightly discoloured effusion. The entire lung also inflamed, and near anterior part gangrenous. Left lung universally and strongly adherent.—*Abdomen:* Liver slightly irregular on surface, and of mottled colour, like nutmeg. Gall bladder small, and filled with concretions. Dropsical effusion in lower part of cavity.

No. 93.—M., æt. 35. In hospital three months.—*Head:* Skull-cap vascular and heavy. Vessels of dura mater turgid. Slight general thickening and opacity of arachnoid, with considerable infiltration of pia mater, so that hemispheres presented perfectly smooth surface, as if covered by whitish-grey gelatinous substance. Choroid plexus pale, and bloodvessels of brain empty. Increased quantity of fluid in ventricles, so as to make opening of communication direct; and much also remained in base of skull after removal of brain.—*Chest:* Several elongated and slender adhesions of left lung.—*Abdomen:* Intense vascular congestion in portion of ilium near termination. Mucous membrane of colon destroyed by ulcerations irregularly over whole extent of bowel, intervening portions being thickened, prominent, and of livid colour.

No. 94.—M., æt. 52. In hospital nine weeks.—*Head:* Vessels of brain slightly turgid, particularly traversing cerebral substance. About an ounce of fluid in lateral ventricles, and nearly an equal quantity in base of skull after removal of brain.—*Chest:* Left lung consolidated, of very dark, almost black, colour, and yielded an offensive odour. Right lung consolidated throughout, with portions so discoloured and fœtid as to appear on point of mortification.

No. 95.—F., æt. 58. In hospital eight months.—*Head:* Vessels on surface of brain distended with blood. Partial opacity of arachnoid. Pia mater infiltrated by serum in every part, which extended down between convolutions. Cerebral substance firm.—*Chest:* Some old adhesions of pleuræ in both cavities. Lungs tuberculous, cut surface solid and mottled. Heart pale.—*Abdomen:* Kidneys large and pale. Corpus laterum in right ovary.

No. 96.—M., æt. 55. In hospital four months and three weeks.—*Head:* Dura mater adhered so firmly to bone that it was torn before brain could be exposed. Large quantity of serous fluid escaped from cranial cavity. Arachnoid slightly opaque. Cerebral substance firm. Ventricles large. Much fluid at base of skull and in vertebral column.—*Chest:* Heart deep red. Ætheromatous earthy deposit on coronary arteries, and considerable hypertrophy of left ventricle.—*Abdomen:* Mucous membrane of large intestine thickened, in parts ulcerated, and in others sloughed.

No. 97.—M., æt. 59. In hospital ten months.—*Head:* Internal vessels congested. Slight infiltration of pia mater. Increased quantity of fluid in lateral ventricles, and about two table-spoonfuls at base of skull after removal of brain.—*Chest:* Both lungs inflamed, and right partly hepatized.—*Abdomen:* Large intestine slightly thickened and extensively ulcerated; mucous membrane of nearly livid colour. Kidneys under natural size.

No. 98.—F., æt. 64. In hospital four months and three weeks.—*Head:* Much blood escaped on opening cranium. Vessels of bone and dura mater turgid. Skull-cap thick, dense, and heavy. Convolutions of cerebral hemispheres shrunken in two or three places; vacuities being occupied by infiltration of pia mater. Much fluid about velum and in base of skull. Yellow ætheromatous deposits to considerable extent in cerebral arteries.—*Chest:* Heart very large, and measured nearly five inches from apex to base. Ventricular valves diseased. Endocardium of left ventricle opaque, thickened, and indurated to whole extent. Adhesion of pericardium to organ. Several ounces of fluid in each pleuræ, and both lungs inflamed.—*Abdomen*: Liver of nutmeg appearance, and kidneys small.

No. 99.—M., æt. 39. In hospital two months.—*Head:* Vessels, both external and internal, empty. Partial opacity of arachnoid covering hemispheres. Some infiltration of pia mater, and augmented quantity of fluid in ventricles.—*Chest:* Both lungs tuberculated, with numerous excavations containing purulent effusions. Right, but especially left, strongly adherent at diseased part.

No. 100.—F., æt. 42. In hospital twelve months.—*Head:* Vessels of skull and membranes loaded with blood. Slight milky opacity of arachnoid on cerebral hemispheres. Considerable infiltration of pia mater. About two ounces of pellucid fluid in each lateral ventricle.

(*To be Continued.*)

